# White emission in 3D-printed phosphor microstructures†

**DOI:** 10.1039/d2cc06953a

**Published:** 2023-02-15

**Authors:** Jędrzej Winczewski, Manuel Herrera, Han Gardeniers, Arturo Susarrey-Arce

**Affiliations:** a Mesoscale Chemical Systems, MESA+ Institute, University of Twente P.O. Box 217 Enschede 7500 AE The Netherlands j.p.winczewski@utwente.nl a.susarreyarce@utwente.nl; b Centro de Nanociencias y Nanotecnología, Universidad Nacional Autónoma de México Km 107 Carretera Tijuana-Ensenada Ensenada Baja California C.P. 22800 Mexico

## Abstract

Microscale functional materials permit advanced applications in optics and photonics. This work presents the additive manufacturing of three-dimensional structured phosphors emitting red, green, blue, and white. The development is a step forward to realizing additive colour synthesis within complex architectures of relevance in integrated optics or light-emitting sources.

## Abstract 2

This work presents the additive manufacturing of 3D phosphors emitting red, green, blue, and white. The development is a step toward additive colour synthesis in complex architectures relevant to integrated optics or light-emitting sources.

## Graphical abstract

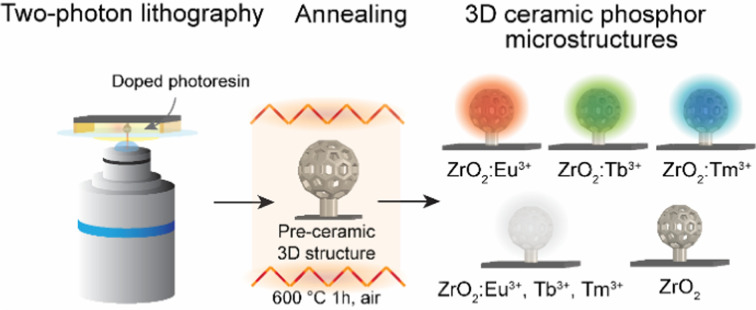

Two-photon lithography (TPL) has unfolded as a buoyant methodology permitting the fabrication of complex three-dimensional (3D) structures with sub-micrometer resolution.^1^ In standard microscope-based TPL systems, femtosecond laser radiation is utilized to initiate the photopolymerization within the focal point, which position is scanned to solidify the negative-tone photoresin into the desired 3D shapes (Fig. 1).^1^ TPL is often used with other fabrication approaches to exploit the full potential, as the intrinsic properties of the standard organic photoresins offer a limited range of applications. Recently, alternatives to standard organic photoresins based on simple monomeric species have been developed. Besides the polymers of modified functionalities, the materials manufactured using the tailor-made photoresins include, *e.g.*, metals, ceramics, or nanocomposites.^2–4^ The pre-ceramic photoresins have reached increasing recognition attributed to their technological importance and the versatile characteristics of the resulting materials, *e.g.*, piezoelectricity, and high mechanical strength.^3,5,6^ Examples of 3D-structured ceramic microstructures include, *i.e.* ZnO, TiO_2_, and ZrO_2_.^3,5,6^ Due to their high chemical and physical stability, resistance to continuous excitation, and high lattice-binding energies, ceramics are often the host matrices of choice for rare-earth ions (RE^3+^).^7^ Upon excitation, phosphorous materials are highly-efficient emitters of narrowly defined radiation that find a broad range of applications, such as microoptics.^8^ Low phonon energy ceramics are typically preferred, as they promote higher RE^3+^ optical transitions quantum efficiencies by reducing non-radiative decay rates.^9^ Sufficient RE^3+^ solubility is required to prevent dopant clustering.^9^ ZrO_2_ fulfils the above considerations and has frequently been applied as a RE^3+^ host. The rationale is that the microscale 3D structuring of phosphors may grant measures for mitigating thermal quenching *via* optimized heat dissipation or restraining the optical crosstalk.^10^ Various forms of structured ZrO_2_ phosphors have been manufactured, *i.e.*, nanotube arrays, nanofibers, or macro-mesoporous structures.^11–13^ Recently, we presented an alternative methodology for fabricating compound 3D ZrO_2_:Eu^3+^ microstructures emitting in orange-red.^10^ The development of 3D phosphors emitting in other primary colours and white is a step forward to realizing additive colour synthesis within complex architectures of relevance in integrated optics or light-emitting sources.^8^ Further, the biocompatibility of ZrO_2_ could also prospectively permit their use in biomedical imaging or sensing.^14,15^

**Fig. 1:**
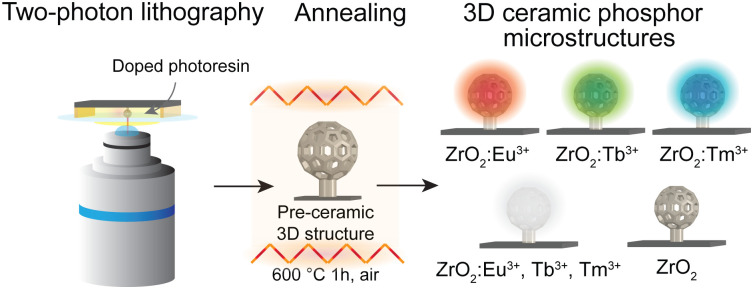
Schematic simplified presentation of the AM of 3D pre-ceramic microstructures using tailor-made photoresists doped with RE^3+^ species and formation of ceramic replicas upon annealing at 600 °C for 1 h.

In this study, we present the additive manufacturing (AM) of ZrO_2_ doped structures emitting in red (Eu^3+^), green (Tb^3+^), blue (Tm^3+^), and white (Eu^3+^, Tb^3+^, and Tm^3+^). The method involves the preparation of tailor-made photoresins suitable for TPL, containing the Zr-rich acrylate monomer and acetate (Ac) salts of the lanthanides (Ln^3+^). TPL is utilized to fabricate 3D architectures of arbitrary shapes from the custom-made photoresin. TPL has a miniaturization advantage, which is also a bottleneck regarding large-scale production. During the printing, the photoresin photopolymerizes, and both organic and metal–organic acrylates participate in the reaction, forming a metal–organic (pre-ceramic) polymer. In the photopolymer, and thus within the printed 3D structure, Ln^3+^ acetates are trapped, similar to the concept presented by Yee *et al.*^3^ During the annealing, the organic constituents of the photopolymer (and the Ac groups of Ln^3+^ acetates) are combusted in the air. As a result, the metal–organic photopolymer is decomposed, the metallic part is oxidized, and the corresponding metal oxide (ZrO_2_) is formed. Trivalent ions (*e.g.*, Ln^3+^) can substitute Zr^4+^ ions in the ceramic matrix and act as dopants. The pre-ceramic architectures (in shape inspired by a C_60_ buckyball) printed on Si substrates are annealed at 600 °C for 1 h. After combustion, the doped ZrO_2_ ceramics (ZrO_2_:Ln^3+^) is obtained, where Ln^3+^ is Eu^3+^, Tb^3+^, or Tm^3+^. Additionally, triply-doped ZrO_2_ is prepared with Eu^3+^, Tb^3+^, and Tm^3+^. The doping is adjusted to 3 wt. % for the RE^3+^ species in ZrO_2_:Ln^3+^, and to 1 wt. % of each RE^3+^ species in the triply-doped material. The methodology is schematically depicted in Fig. 1. and described in the ESI† (S1.2).

The annealing promotes the formation of miniaturized ceramic replicas isotropically reduced in size by ≈ 60%. The result is shown in Fig. 2(a). Complimentary images of ZrO_2_:Tb^3+^ and ZrO_2_:Tm^3+^ gyroids and a ZrO_2_:Eu^3+^, Tb^3+^, Tm^3+^ octet-truss lattice can be found in ESI† (Fig. S1). In Fig. 2(b–g), elemental mapping is conducted using Scanning Electron Microscopy-Energy Dispersive X-ray Spectroscopy (SEM-EDX), which confirms the uniform distribution of co-dopants throughout the ZrO_2_ architecture. The O and Si signals are absent within the structural beams, and surface oxide on the Si substrate is observed. The EDX spectrum (Fig. 2(i) features sharp O Kα_1_, Si Kα_1_ (substrate), and Zr Lα peaks centred at 0.52 eV, 1.74 eV, and 2.12 eV.^16^ The insets present the signals originating from the RE^3+^ species. The Eu contributions are found at 1.14 eV (Mα_1_), 5.85 eV (Lα_1_), and 6.46 eV (Lβ_1_).^16^ Characteristic Tb peaks at 1.24 eV (Mα_1_), 6.27 eV (Lα_1_), and 6.98 eV (Lβ_1_), and Tm signals at 1.46 eV (Mα_1_), 7.18 eV (Lα_1_), and 8.10 eV (Lβ_1_) are also detected.^16^ The analysis confirms the incorporation of Eu, Tb, and Tm within the ZrO_2_ structure. The site symmetry and structure influence the radiative transitions of the hosted RE^3+^ ions.^17^ The crystallographic phase of the undoped and doped ZrO_2_ is assessed with X-ray diffraction (XRD) (Fig. S3, ESI†) and cross-checked with confocal Raman spectroscopy (Raman) (Fig. S4, ESI†). In short, for XRD and Raman, tetragonal zirconia (*t*-ZrO_2_) is observed for the doped ZrO_2_. The estimated crystallite sizes are approximately 7 nm (S.4.1., ESI†). Four intense Raman modes are detected at 145 cm^−1^ (*B*_1g_), 267 cm^−1^ (*E*_g_), 462 cm^−1^ (*E*_g_), and 646 cm^−1^ (*E*_g_) with weaker shoulders at 316 cm^−1^ (*B*_1g_), and 606 cm^−1^ (*B*_1g_) for the undoped *t*-ZrO_2_ microstructure.^18–20^ All these vibrational modes are also observed for ZrO_2_ doped with Eu^3+^, Tb^3+^, and Tm^3+^, and ZrO_2_ co-doped with Eu^3+^, Tb^3+^, and Tm^3+^. The results agree with the literature on ZrO_2_ doped with ≈ 3 wt% of Ln^3+^ species and with our previous work.^10,21^ In a previous study employing X-ray photoelectron spectroscopy, we confirmed the predominant oxidation state of Eu species introduced into the ZrO_2_ microstructures.^10^ In the triply-doped ZrO_2_, the Ln^3+^ loadings are low (≈ 1%), making it challenging to determine the species. Thus, we rely on the optical properties of the 3D structured ZrO_2_, ZrO_2_:Ln^3+^, and ZrO_2_:Eu^3+^, Tb^3+^, Tm^3+^ phosphors.

**Fig. 2:**
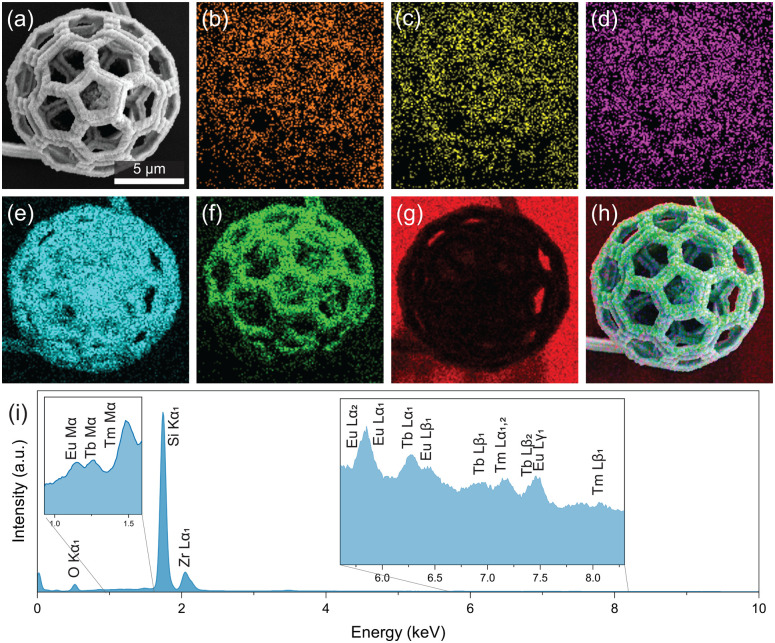
(a) SEM image of the triply-doped ZrO_2_ buckyball; (b–g) SEM-EDX elemental maps of (b) Eu, (c) Tb, (d) Tm, (e) Zr, (f) O, (g) Si, and (h) overlayed (a–g) images. (i) EDX spectrum collected from the ZrO_2_:Ln^3+^ buckyball.

The optical properties of the 3D phosphor structures are investigated with cathodoluminescence (CL). Although differences in photoluminescence (PL) and CL spectra may occur, the microscale feature size of the architectures is the main reason for selecting the CL in this study.^22^ The CL spectrum collected from the undoped *t*-ZrO_2_ buckyball is a sum of Gaussian components at approximately 3.5 eV (356 nm), 3.2 eV (393 nm), 2.9 eV (434 nm), 2.6 eV (485 nm), and 2.3 eV (546 nm) (Fig. 3(a)), previously assigned to the F^+^ centers, oxygen vacancies (*V*_O_) and interstitial carbon (*C*_i_) point-defects.^10^ The spectra of ZrO_2_ with a single RE^3+^ ion type reveal their characteristic transitions, significantly more intense than the negligible *t*-ZrO_2_ CL components.^10^ The CL spectrum of ZrO_2_:Eu^3+^ is dominated by the sharp ^5^D_0_ → ^7^F_1_ and ^5^D_0_ → ^7^F_2_ (594 nm and 609 nm) and weak ^5^D_0_ → ^7^F_2_ (≈628 nm) and ^5^D_0_ → ^7^F_3_ (≈651 nm) transitions (Fig. 3(b)) are registered. The CL spectrum of ZrO_2_:Tb^3+^ features the sharp ^5^D_4_ → ^7^F_5_ (546 nm) line, medium-intensity ^5^D_4_ → ^7^F_6_ (491 nm) emission, and weak ^5^D_4_ → ^7^F_J_ (*J* = 4, 3) peaks (590 nm, 622 nm) (Fig. 3(c)).^23^

**Fig. 3:**
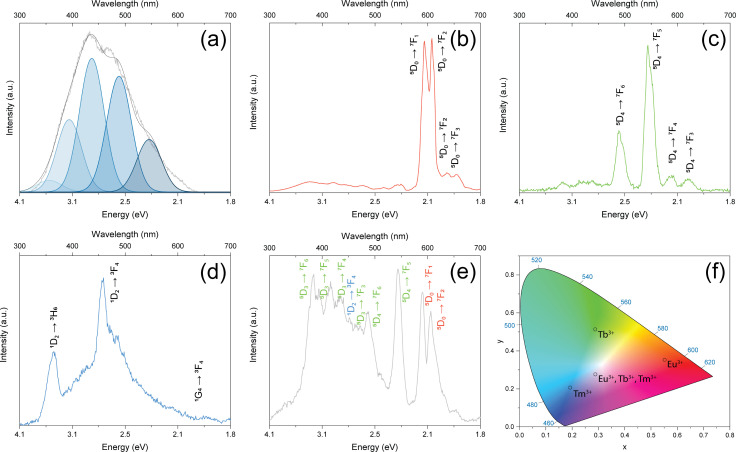
CL spectra registered for the buckyballs: (a) ZrO_2_, (b) ZrO_2_:Eu^3+^, (c) ZrO_2_:Tb^3+^, (d) ZrO_2_:Tm^3+^, and (e) ZrO_2_ triply-doped with Eu^3+^, Tb^3+^, and Tm^3+^, and (f) 1931 CIE chromaticity diagram with labels corresponding to the RE^3+^ dopants in ZrO_2_.

In the violet-blue spectral region, very weak emissions corresponding with ^5^D_3_ → ^7^F_J_ transitions are detected (≈ 378 nm, 414 nm, and 436 nm).^23^ The CL spectrum of ZrO_2_:Tm^3+^ exhibits intense ^1^D_2_ → ^3^F_4_ (459 nm), a medium intensity ^1^D_2_ → ^3^H_6_ line (359 nm), and a very weak ^1^G_4_ → ^3^F_4_ (≈ 648 nm) transition (Fig. 3(d)).^21,24^ The co-doping of ZrO_2_ with Eu^3+^, Tb^3+^, and Tm^3+^ results in distinctive individual contributions and signals related to energy transfer (ET) interactions (Fig. 3(e)). The sharp Eu^3+ 5^D_0_ → ^7^F_1_ (≈590 nm) and ^5^D_0_ → ^7^F_2_ (≈ 606 nm) emission lines, and weaker ^5^D_0_ → ^7^F_2_ (≈ 626 nm) and ^5^D_0_ → ^7^F_3_ (≈ 651 nm) transitions. The distinct Tb^3+ 5^D_4_ → ^7^F_5_ and ^5^D_4_ → ^7^F_6_ transitions are also observed. Several contributions can be assigned within the 360–460 nm region, including the overlapped Tm^3+ 1^D_2_ → ^3^F_4_ (459 nm) transition. The sharp Tm^3+^ emissions cannot unambiguously be distinguished.^21^ The suppression of the Tm^3+ 1^D_2_ → ^3^H_6_ transition (observed around 359 nm in the ZrO_2_:Tm^3+^ spectrum) and accompanying appeareance of Tb^3+ 5^D_3_ → ^7^F_J_ emissions may imply the ET from the Tm^3+ 1^D_2_ level to Tb^3+ 5^D_3_ level, likely involving a relaxation.^21,22,25^ Consistently, several intense Tb^3+^ transitions (^5^D_3_ → ^7^F_6,5,4,3_) are observed (≈ 380 nm, 410 nm, 440 nm, and 460 nm), which have previously been reported at low Tb^3+^ (≈ 2 at%) concentrations in LiLuF_4_ under 353 nm excitation.^22^ At lower Tb^3+^ concentrations, the probability of cross-relaxation decreases, promoting the ^5^D_3_ → ^7^F_J_ transitions.^26^ The effect is significant at low concentrations in the case of CL spectra but not observed in the case of PL.^26,27^ The blue emission has been proposed to be correlated with the presence of Tb^4+^ ions, which can substitute the Zr^4+^ ion positions. Upon the impact of electrons from the beam during CL measurements, Tb^4+^ ions can transiently be excited to the (Tb^3+^) state and act as emissive centers.^27^ Also, some of the excited electrons from the Tb^3+ 5^D_4_ level may cross-relax to the Eu^3+ 5^D_0_ level and contribute to the ^5^D_0_ → ^7^F_0_, 1, 2 transitions.^21^ We observe that the intensity of the ^5^D_0_ → ^7^F_1_ transition is higher than the ^5^D_0_ → ^7^F_2_ in the triply-doped sample. In principle, such an ET is very efficient due to the overlap of the Tm^3+ 5^D_4_ → ^7^F_6,5,4,3_ emissions and the Eu^3+ 7^F_0_,_1_ → ^5^D_0_, 1, 2 absorptions.^21^ The PL from the RE^3+^ is indirectly confirmed by exciting it at 532 nm, using Raman system and Eu^3+^ as an example. A similar concept has previously been presented by Tiseanu *et al*. (Fig. S5, ESI†).^28^

The CL spectra are converted into the Commission Internationale de l’éclairage (CIE) 1931 color space chromaticity diagram (Fig. 3(f)). The CIE coordinates might slightly differ from their respective PL emissions, as the ET may be less apparent in the case of the electron beam excitation when compared with the ultraviolet pump.^22^ The CIE coordinates of single-doped ZrO_2_ fall into the orange-red (Eu^3+^), green (Tb^3+^), and blue (Tm^3+^) regions. The triple doping (Eu^3+^, Tb^3+^, and Tm^3+^) promotes white emission (Fig. 3(f)). Although the main goal of this study is to present the white emission in triply-doped microstructures, additional insights into the effect of high-temperature annealing (1200 °C for 1 and 3 h) on the optical properties of ZrO_2_:Eu^3+^, ZrO_2_:Tb^3+^, ZrO_2_:Tm^3+^, and ZrO_2_:Eu^3+^, Tb^3+^, Tm^3+^ architectures are provided (Fig. S6, ESI†). Complimentary SEM images of the buckyballs annealed at 1200 for 3 h are also presented (Fig. S2, ESI†). The panchromatic CL image (Fig. 4(b)) presents the homogenous emission from the buckyball. The monochromatic CL images (Fig. 4(c–h)) are obtained at 3.24 eV, 2.99 eV, 2.55 eV, 2.28 eV, 2.10 eV, 2.05 eV to match the most intense transitions registered in the triply-doped sample, corresponding to the 383 nm, 415 nm, 486 nm, 544 nm, 594 nm, and 605 nm in the visible range. Interestingly, CL images obtained at 3.24 eV, 2.99 eV, and 2.55 eV correspond to the transitions occurring mainly due to the ET, *e.g.*, ^5^D_3_ → ^7^F_6_, or ^5^D_3_ → ^7^F_5_, in which more uniform emission throughout the architecture is observed. Brighter regions are observed mainly at the edges of the architecture in the CL images obtained at 2.28 eV, 2.10 eV, and 2.05 eV, indicating possible Ln^3+^ segregation. Nevertheless, these regions can be associated with the most intense ^5^D_4_ → ^7^F_5_ transition of ZrO_2_:Tb^3+^ and ^5^D_0_ → ^7^F_1_ and ^5^D_0_ → ^7^F_2_ transitions of ZrO_2_:Eu^3+^.

**Fig. 4:**
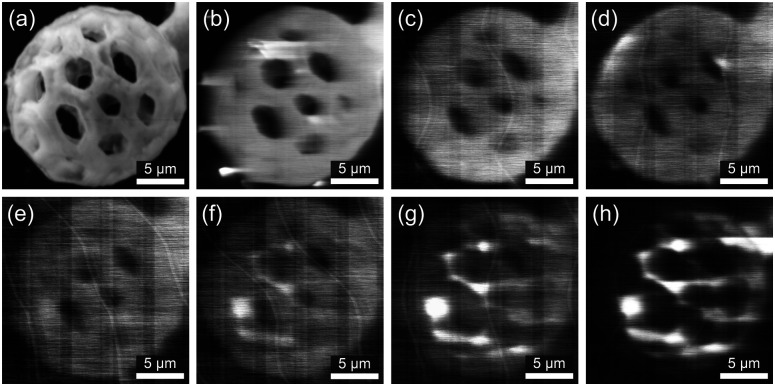
Micrographs of the ZrO_2_ buckyball doped with Eu^3+^, Tb^3+^, and Tm^3+^; (a) SEM secondary electron image, (b) SEM panchromatic CL image, (c–h) monochromatic CL images at (c) 3.24 eV, (d) 2.99 eV, (e) 2.55 eV, (f) 2.28 eV, (g) 2.10 eV, and (h) 2.05 eV. Scale bars represent 5 μm.

In summary, we present tailor-made photoresins permitting the AM of microstructures doped with RE^3+^ species (Eu^3+^, Tb^3+^, Tm^3+^) *via* TPL and subsequent annealing in the air. The thermal treatment of the 3D pre-ceramic structures results in forming ceramic replicas that are isometrically reduced in size by ≈60%. The incorporation of the RE^3+^ species within the ZrO_2_ host is confirmed *via* SEM-EDX imaging. The *t*-ZrO_2_ crystallographic phase of the fabricated ZrO_2_:Ln^3+^ microstructures is confirmed using Raman spectroscopy. The coherent results are backed by XRD analysis of the reference ceramic powders obtained from the UV-cured photoresins annealed in bulk. The transitions of the ZrO_2_:Ln^3+^ 3D structures are evaluated using CL, and according to the 1931 CIE colour space chromaticity diagram, the green (ZrO_2_:Tb^3+^), and blue (ZrO_2_:Tm^3+^) emissions are confirmed. The triple-doping promotes the ET from the Tm^3+^ to Tb^3+^ and from the Tb^3+^ to Eu^3+^, and consequently, the white emission. The results show that the presented approach is suitable for the realization of complex 3D microarchitectures emitting close to all primary colours, and white.

J.P.W., A.S.-A., and H.G. are recipients of the Horizon 2020 ERC research and innovation programme of the European Union funding under Grant Agreement No. 742004. M.H. acknowledges support from the University of California Institute for Mexico and The United States (UCMEXUS) (No. CN19137) and CONACYT (Grant No. 284667).

## Conflicts of interest

There are no conflicts to declare.

## Supplementary Material

CC-059-D2CC06953A-s001
