# Supplementary material for: White emission in 3D-printed phosphor microstructures
Source: Chem Commun (Camb). 2023 Feb 15;59(21):3095–8. doi: 10.1039/d2cc06953a (PMC9997107; doi:10.1039/d2cc06953a)
Supplement: CC-059-D2CC06953A-s001 [file CC-059-D2CC06953A-s001.pdf]

Electronic Supplementary Material (ESI) for Chemical Communications.
This journal is © The Royal Society of Chemistry 2023

Electronic Supplementary Information for

White emission in 3D-printed phosphor microstructures

Jędrzej Winczewski,[a] Manuel Herrera,[b] Han Gardeniers,[a] and Arturo Susarrey-Arce,[a]

[a.] *Mesoscale Chemical Systems MESA+ Institute University of Twente P.O. Box 217, Enschede 7500 AE, The Netherlands.*

[b.] *Centro de Nanociencias y Nanotecnología Universidad Nacional Autónoma de México Km 107 Carretera Tijuana-Ensenada, Ensenada Baja California C.P. 22800, Mexico C.*

S1. Experimental Section

S.1.1. Materials

All agents were used as received without additional purification. Acrylic acid (99,9%, stabilized with ca 200 ppm 4-methoxyphenol; AA), dichloromethane (99.9 %; DCM), europium triacetate hydrate (99.99%; EuAc x H_2O), terbium triacetate hydrate (99%; TbAc x H_2O), and thulium triacetate hydrate (≥99.9%; TmAc x H_2O) were purchased from Alfa Aesar. Dimethylsulfoxide (≥99.9%; DMSO), 2-propanol (≥99.5%; IPA) N,N-dimethylacetamide (anhydrous, 99.8%; DMAc), pentaerythritol triacrylate (technical grade; PETA), and zirconium acrylate (ZrA were ordered from Sigma-Aldrich. J&K Scientific delivered 7-Diethylamino-3-thenoylcoumarin (97%, DETC).

S.1.2. Additive manufacturing

S.1.2.1. Photoresin preparation

In total, four different photoresins permitting the additive manufacturing of ZrO_2 doped with Eu^{3+}, Tb^{3+}, and Tm^{3+} and co-doped with Eu^{3+}, Tb^{3+}, and Tm^{3+} are tailor-made. The process consists of two steps, i.e., preparation of the dopant solution and formulation of the photoresin base, which components are later combined. To reduce UV exposure, the photoresin and its components were handled under yellow lightning.

The preparation of Eu^{3+}, Tb^{3+}, and Tm^{3+} doping and co-doping solutions

In 3 ml amber glass vials, lanthanide salts (Table S1.) are combined with DMAc to obtain a total weight of 1000 mg using the analytical microbalance. For Eu^{3+} doping, EuAc x H_2O (6.59 mg, 20.02 µmol) is used; for Tb^{3+} doping, TbAc x H_2O (6.44 mg, 19.15 µmol) is taken; and for Tm^{3+} doping, TmAc x H_2O (6.24 mg, 18.02 µmol) is applied. The anhydrous basis is taken as a reference; thus, the actual doping levels are lower in practice. For the co-doped sample containing Eu^{3+}, Tb^{3+}, and $Tm^{3+,}$ all the salts mentioned above are used (2.2 mg EuAc x H_2O, 6.68 µmol; 2.15 mg TbAc x H_2O, 6.38 µmol; 2.08 mg

TmAc x H_2O, 6.00 µmol). The contents are dissolved using a vortex mixer for 5 minutes and later placed in a water bath set at 40 °C for one hour to obtain transparent solutions.

The preparation of Zr-containing lanthanide-doped photoresin

In a 50 ml amber round bottom flask, ZrA (75 mg, 0.2 mmol) was dissolved in 250 mg of DMAc (250 mg, 2.87 mmol) and DCM (250 mg, 2.94 mmol) by mixing the contents for 1 hour in a rotary evaporator operated under ambient pressure at 40 °C, and 150 rpm. Then, PETA (300 mg, 1.01 mmol) and 250 mg of the doping or co-doping solution were added, and the rotation-mixing resumed for one hour. DETC (23 mg, 76.8 µmol) and DCM (100 mg, 1.18 mmol) were transferred to the mixture. After the dissolution of the photoinitiator, the pressure was reduced for 30 minutes to remove DCM completely. The obtained photoresin was pipetted to the storage vial and used directly.

S 1.2.2. Two-photon lithography

The pre-ceramic microstructures were written using tailor-made photoresins, and a direct laser writing system (Photonic Professional GT, Nanoscribe) operated in the reflection mode. In the setup differential interference contrast, 63×/1.4 NA objective (Plan-Apochromat, Carl Zeiss) immersed in oil (Immersol 518 F, Carl Zeiss) focused the femtosecond laser radiation on the interface between a silicon dice (1 x 1 cm) and the photoresin. The polished reflective side of the Si dice was placed on parallel ribbons of a double-sided polyimide adhesive tape, facing the objective. The 3D structures were patterned using the laser set at 18.9 mW power and 1 mm s^{-1} scanning. After printing, the detached Si dices were immersed in DMSO for 2 minutes, IPA for 2 minutes to develop, and later placed for 12 hours in a drying oven set at 65 °C.

S 1.2.3. The preparation of the bulk-cured photoresin

To bulk-cure the photoresin, it was pipetted into a glass Petri dish and cured with ultraviolet (UV) radiation (36W, 365 nm) using a commercial desktop lamp (EBN001, Esperanza). After 3 hours of exposure, the thin film was pulverized and placed in a drying oven (Model ED 23, Binder) at 80 °C for 12 hours to remove the potential volatile component traces. The dried photopolymer was later used to synthesize the bulk reference ceramic powder.

S 1.2.4. Thermal processing

The Si dices with the 3D-printed pre-ceramic architectures and bulk-cured photopolymers were placed in Alsint® crucibles and annealed in a chamber oven (LH 15/12, Nabertherm) with opened air inlets. The 1 °C min^{-1} heating ramp was first set to reach 500 °C, and then 2 °C min^{-1} to attain 600 °C, which temperature was maintained for one hour. The oven was then cooled at a natural ramp. The annealing resulted in the combustion of the organic constituents of the photocured materials and promoted the formation of the target doped ceramics.

S.2. Characterization

S.2.1. Characterization of the 3D ceramic architectures

S.2.1.1. Cathodoluminescence (CL)

The cathodoluminescence spectra and images were collected at ambient temperature with a Gatan MonoCl4 detector using a JEOL JIB-4500 scanning electron microscope operated at 15 kV acceleration voltage. The CL spectra were converted from the photon energy (eV) to the corresponding wavelength (nm) and processed using OriginPro 2022 software to generate the Commission Internationale de l'éclairage (CIE) 1931 color space chromaticity diagram.

S.2.1.2. Confocal Raman Spectroscopy (Raman)

Upon excitation with a 532 nm laser set at 5.0 mW power, the Raman spectra were collected at room temperature with a commercial Raman microscope (Alpha 300, WiTec) in standard backscattering geometry. An air objective (100×/0.9 NA, MPlan FL N, Olympus) and 600 g mm^{-1} grating were used. The presented spectra were collected for half a second and are the average of 100 acquisitions. The data was processed to remove the cosmic rays and baseline-corrected with freeware software (CrystalSleuth, RRUFF™ Project).[1]

S.2.1.3. Scanning Electron Microscopy (SEM) and Energy-Dispersive X-ray Spectroscopy (EDX)

The electron images of the 3D architectures were registered with Inlens, and High-Efficiency Secondary Electron detectors of the SEM station (Carl Zeiss, Merlin AURIGA CrossBeam Workstation) operated at 1.4 kV acceleration voltage. The energy-dispersive X-ray spectrum and maps showing the elemental distribution throughout the architecture were acquired using the 15 kV acceleration voltage using the AZtec (Oxford Scientific) application. The Secondary Electron SEM images were acquired using a JEOL JIB-4500 SEM operated at 15 kV and 7 kV acceleration voltage.

S.2.1.4. X-Ray Powder Diffraction (XRD)

The X-Ray Powder patterns of the reference powders were registered using a LynxEye detector of the desktop diffractometer (D2 Phaser, Bruker) with a Cu-Kα source (λ = 1.5406 Å) operated at 30 kV and 10 mA. The diffractograms of the samples cast on the zero-diffraction substrate were collected within the 2θ 20°–80° scan range with a 2.25° min^{-1} scan speed. The baseline correction was performed using a default algorithm of freeware software (CrystalSleuth, RRUFF™ Project).[1]

S.3. Supplementary images

S.3.1. Supplementary SEM images of microstructures

In Fig. S5., supplementary SEM and panchromatic CL images of additional 3D structure designs, i.e., ZrO_2:Tb^{3+} and ZrO_2:Tm^{3+}, and ZrO_2:Eu^{3+} Tb^{3+} Tm^{3+} octet-truss lattice annealed at 600 °C for 1h, are presented.

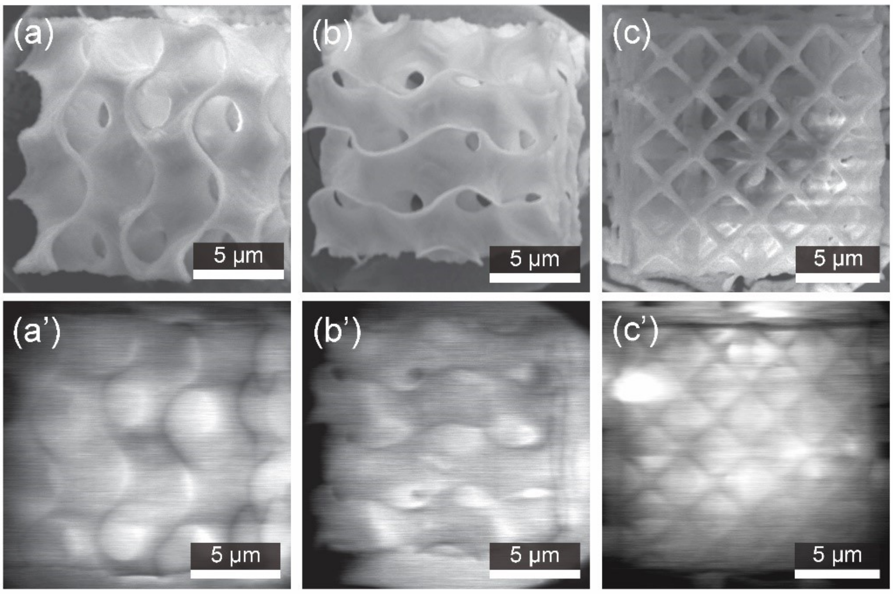

Fig. S1 (a – c) SEM and (a' – c') panchromatic CL images of samples annealed at 600 °C for 1 h: (a, a') ZrO_2:Tb^{3+} gyroid, (b, b') ZrO_2:Tm^{3+} gyroid, (c, c') ZrO_2:Eu^{3+}, Tb^{3+}, Tm^{3+} octet-truss lattice. Scale bars represent 5 μm.

In Fig. S6, additional SEM images of (a) ZrO_2:Eu^{3+}, (b) ZrO_2:Tb^{3+}, (c) ZrO_2:Tm^{3+}, and (d) ZrO_2:Eu^{3+}, Tb^{3+}, Tm^{3+} buckyballs annealed at 1200 °C for 3 h are given. Fig. S6 (a' – d') shows high-magnification SEM images of the areas indicated with boxes in images (a – d). The presence of crystalline grains is confirmed. The sintering of crystallites is pronounced at the edges and may result in structural deformations.

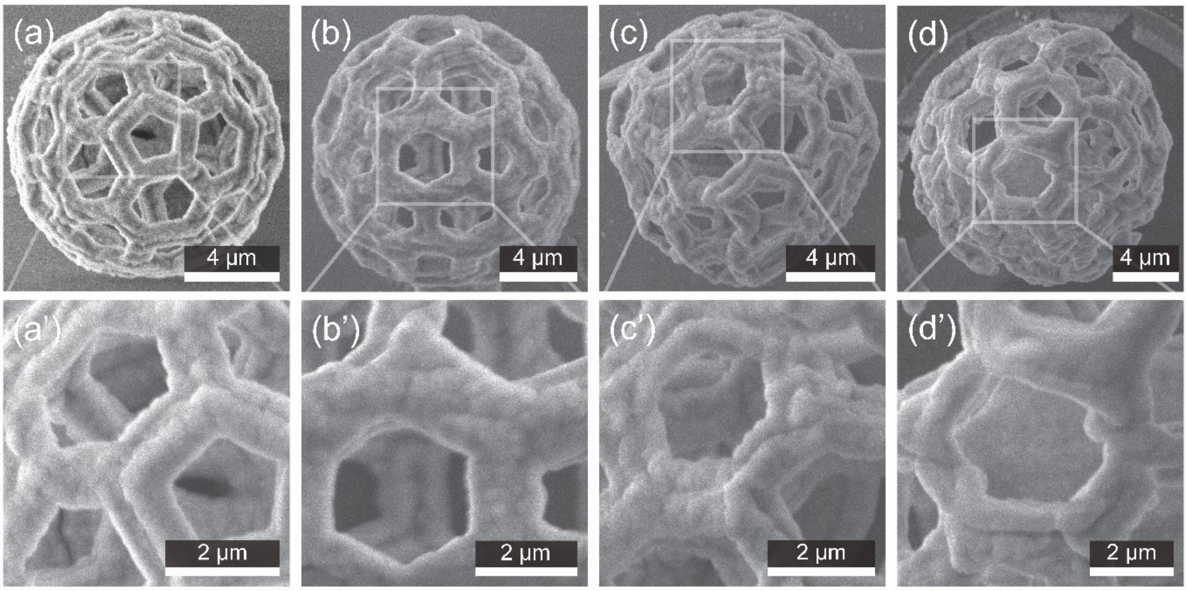

Fig. S2 SEM images of buckyballs annealed at 1200 °C for 3 h: (a) ZrO_2:Eu^{3+}, (b) ZrO_2:Tb^{3+}, (c) ZrO_2:Tm^{3+}, and (d) ZrO_2:Eu^{3+}, Tb^{3+}, Tm^{3+}. (a' – d') high-magnification SEM images of areas indicated with boxes in images (a – d). Scale bars represent (a – d) 4 µm and (a' – d') 2 µm.

S.4. Supplementary spectra

S.4.1. XRD patterns of reference ceramic powders

The ZrO_2 doping with trivalent species results in the formation of V_O, compensating for the charge and promoting the sevenfold coordination of the Zr cations.[2] Under the conditions applied in this study, a tetragonal phase is expected. The XRD patterns of the ZrO_2, ZrO_2:Eu^{3+}, ZrO_2:Tb^{3+}, ZrO_2:Tm^{3+}, and ZrO_2:Eu^{3+}, Tb^{3+}, Tm^{3+} reference powders are presented below in Figure S2. All the diffractograms are distinct of the tetragonal zirconia (t-ZrO_2) phase of the $P4_2/nmc$ space group, with the most intense diffraction at 30.3° (101), and weaker signals around 35.0° (110), 50.3° (112), and 59.9° (101), consistent with the results previously observed for ZrO_2, and ZrO_2:Eu^{3+}, Tb^{3+}, Tm^{3+} at similar RE^{3+} concentrations.[3]

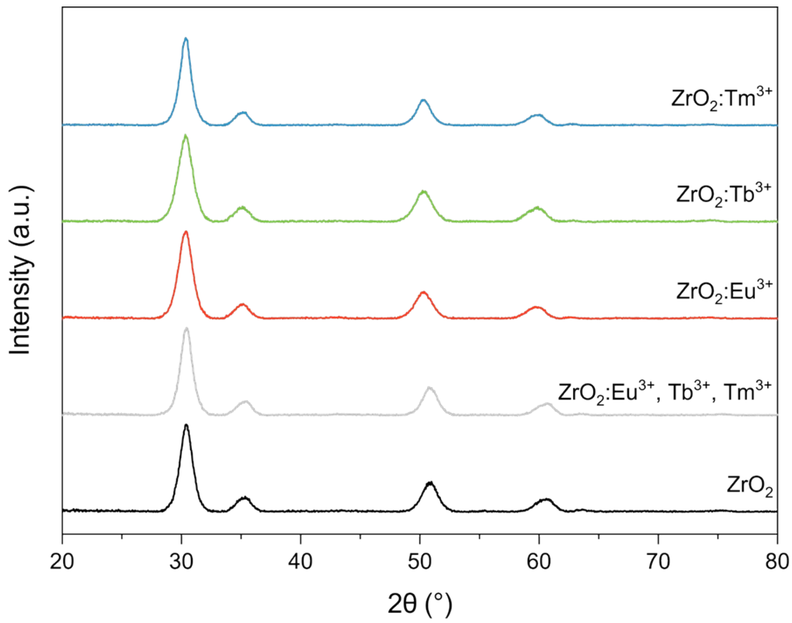

Fig. S3 XRD diffractograms of the ZrO_2, $ZrO_2:Eu^{3+}$, $ZrO_2:Tb^{3+}$, $ZrO_2:Tm^{3+}$, and $ZrO_2:Eu^{3+}$, Tb^{3+}, Tm^{3+} reference powders.

Scherrer equation (1) is used to compute the average size of crystallites (D), based on the used X-ray wavelength (λ = 1.5406 Å), using Scherrer constant, K of 0.89, and the determined full width at half maximum, (FWHM, denoted m) for the measured diffraction angle (θ).[4,5]

$$m = \frac{K\lambda}{D\cos(\theta)} \quad (1)$$

The FWHM is determined using the Gaussian function and OriginPro® 2022 software for the peaks centred around 30.3° (101), 35.0° (110), 50.3° (112), and 59.9° (101). The averaged crystallite sizes are 6.6 ± 0.7 nm for ZrO_2, 7.1 ± 0.4 nm for $ZrO_2:Eu^{3+}$, 6.2 ± 0.4 nm for $ZrO_2:Tb^{3+}$, 7.3 ± 0.5 nm for $ZrO_2:Tm^{3+}$, and 6.5 ± 0.4 nm for triply-doped sample.

S.4.2. Raman spectra of the microstructures

Characteristic Raman vibrational modes of tetragonal ZrO_2 (t-ZrO_2) are registered around 145 cm^{-1} (B_{1g}), 267 cm^{-1} (E_g), 462 cm^{-1} (E_g), and 646 cm^{-1} (E_g). Additional weaker shoulders around 316 cm^{-1} (B_{1g}), and 606 cm^{-1} (B_{1g}), typical t-ZrO_2 are also observed for the undoped microstructure.[6–8] The same Raman modes are observed for ZrO_2 doped with Eu^{3+}, Tb^{3+}, Tm^{3+}, and the triply-doped ZrO_2. The results are consistent with the literature on the ZrO_2 doped with ~3 wt. % of Ln^{3+} species, and our previous study.[3,9]

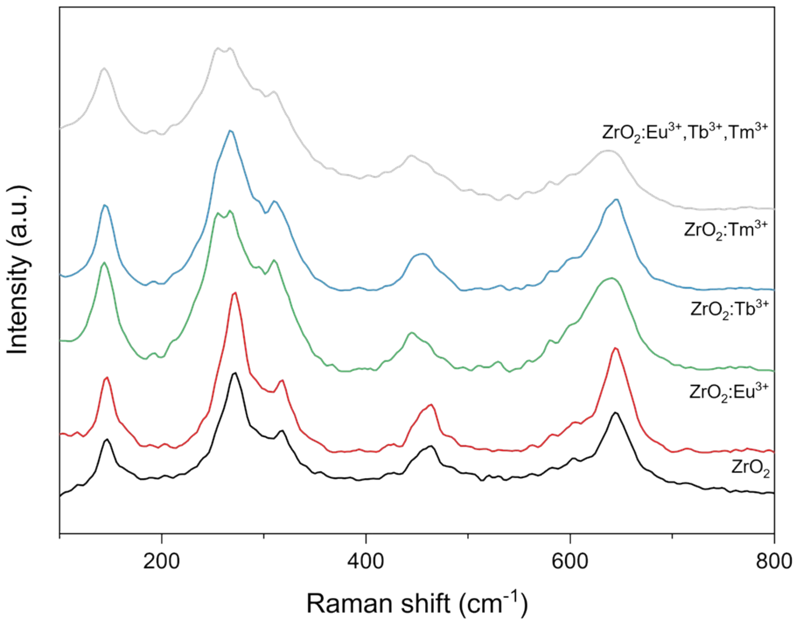

Fig. S4 Confocal Raman spectra registered for the ZrO_2, ZrO_2:Eu^{3+}, ZrO_2:Tb^{3+}, ZrO_2:Tm^{3+}, buckyballs and ZrO_2 microstructure co-doped with Eu^{3+}, Tb^{3+}, and Tm^{3+}.

We consider that using a Confocal Raman Microscope with a green (532 nm) light source at 10 mW to prevent the detector saturation, it is possible to excite the ZrO_2 microstructure doped with Tm^{3+}, $Tb^{3+,}$ and Eu^{3+}. The 532 nm laser has the highest photon energy available in the system (532 nm, 633 nm, and 785 nm). As the emission of Tm^{3+} and Tb^{3+} typically falls below this wavelength, only Eu^{3+} emissions may be observable. A concept has previously been presented by Tiseanu *et al.*[10,11] The spectrum is obtained by focusing into a buckyball toward the substrate (and thus defocusing from the surface). Weak Raman scattering related to the *t*-ZrO_2 lattice and Si peaks is observed in the lower reciprocal lengths in Figure S3.[6–8,12] At the higher wavenumbers, emissions resembling the photoluminescence spectrum of *t*-ZrO_2:Eu^{3+} are observed.[13,14] The Raman spectrum wavelength (λ_1) collected upon excitation at the wavelength (λ_0) can be estimated for the known Raman shift (ν) by converting the wavenumber (cm^{-1}) to wavelength (nm).[15] The following formula estimates the Raman spectrum wavelengths:

$$\nu = \frac{1}{\lambda_0} - \frac{1}{\lambda_1}$$

The ZrO_2:Eu^{3+} transitions are labelled in Figure S3. The most intense signal is related to the $^5D_0 \rightarrow {}^7F_2$ transition, in which splitting into sublevels around 2315 cm^{-1} (607 nm), 2443 cm^{-1} (611 nm), and 2780 cm^{-1} (624 nm) is observed.[14] A weaker $^5D_0 \rightarrow {}^7F_1$ transition, peaked at 1890 cm^{-1} (~591-592 nm) with 1760 cm^{-1} shoulder (587 nm), is also registered. A weak peak related to the $^5D_0 - {}^7F_0$ transition is found at 1505 cm^{-1} (578 nm). [13,14] Finally, a contribution around 3440 cm^{-1} (651 nm), associated with a $^5D_0 \rightarrow {}^7F_3$ transition, is observed.[13,14]

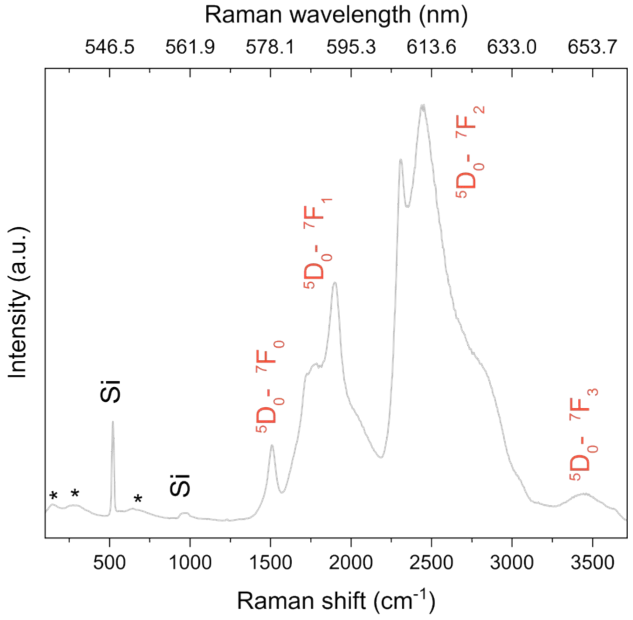

Fig. S5 Confocal Raman spectrum collected from a ZrO_2 microstructure co-doped with Eu^{3+}, Tb^{3+}, and Tm^{3+}, excited at 532 nm, showing characteristic emissions for Eu^{3+} species in the ~1500 – 3700 cm^{-1} range. The specific Eu^{3+} transitions are assigned. The *t*-ZrO_2 Raman vibrational modes are labeled with asterisks (*), and the peaks related to the substrate are labeled as Si.

S.4.3. Supplementary CL measurements

The main goal of this study is to present the realization of white emission in triply-doped (ZrO_2:Eu^{3+}, Tb^{3+}, Tm^{3+}) microstructures. However, to provide additional insights into the effects of the temperature treatment, ZrO_2:Eu^{3+}, ZrO_2:Tb^{3+}, ZrO_2:Tm^{3+}, and ZrO_2:Eu^{3+}, Tb^{3+}, Tm^{3+} microstructures already annealed at 600 °C for one hour are additionally thermally treated at 1200 °C for one and three hours. The annealing is conducted under low pressure (1 mTorr) in an 80:20 mixture of N_2 and O_2 to simulate air. The temperature is chosen, as at 1200 °C monoclinic phase (*m*-ZrO_2) is yielded, and the two different times are selected to investigate whether the changes in spectra are related to the treatment time.[9] Cathodoluminescence spectra collected for these samples are presented below in Fig. S4, in which transitions are assigned to the observed peaks.

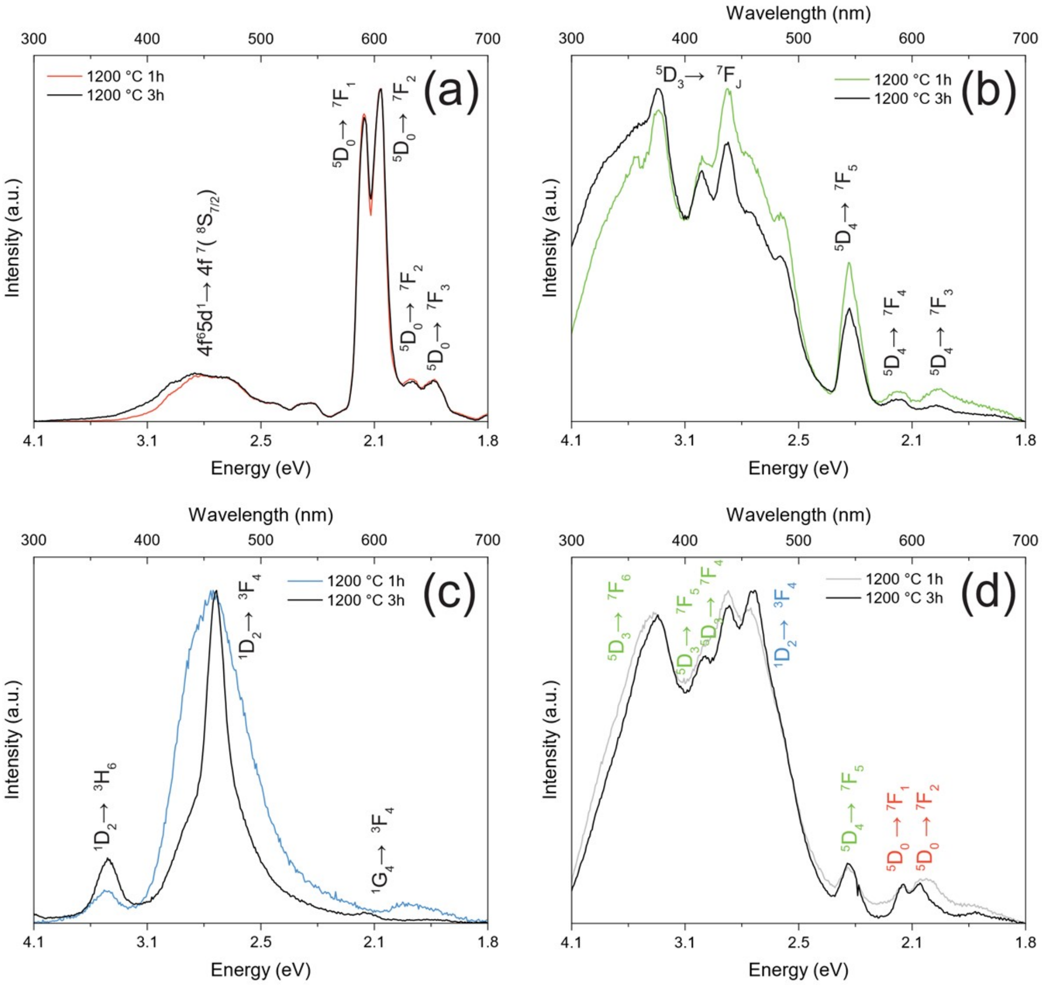

Fig. S6 Cathodoluminescence spectra registered for 3D printed structures annealed at 1200 °C for one and three hours: (a) ZrO_2:Eu^{3+}, (b) ZrO_2:$Tb^{3+,}$ (c) ZrO_2:Tm^{3+}, and (d) ZrO_2:Eu^{3+}, Tb^{3+}, Tm^{3+}. The solid red, green, or red line represents the one-hour treatment, and the black line represents the three-hour treatment.

In the case of ZrO_2:Eu^{3+}, the $^5D_0 \rightarrow {}^7F_J$ (J=1,2,3) transitions are observed, though the $^5D_0 - {}^7F_2$ transition shows relatively higher intensity, in accordance with our previous observations (Fig. S4 (a)).[9] Additional broadband component corresponding to blue-green emissions (≈ 400 – 500 nm) is observed and assigned to the $4f^65d^1 \rightarrow 4f^7$ ($^8S_{7/2}$) Eu^{2+} transition, indicating the partial reduction of Eu^{3+} species to Eu^{2+}. No significant differences are noticed for a sample annealed at 1200 °C for three hours. Previously, we observed a partial reduction of Eu^{3+} to Eu^{2+} after similar thermal treatment in the ZrO_2:Eu^{3+} XPS data collected for reference powder.[9]

Next, CL spectra registered for ZrO_2:Tb^{3+} are analysed. Significant differences are noted, correlated with the complex changes within the sample state, as indicated with the assigned $^5D_3 \rightarrow {}^7F_J$ and $^5D_4 \rightarrow {}^7F_J$ transitions (Fig. S4 (b)), which origin is briefly discussed.[16] The relative intensity of the blue and

green Tb^{3+} $^5D_3$ and $^5D_4$ level emissions are correlated with the Tb^{3+} concentration.[17] The energy difference between the Tb^{3+} $^5D_3$ and $^5D_4$, and $^7F_0$ and $^7F_6$ levels, is similar, which renders the emissions related to the $^5D_3$ level strongly dependent on the cross-relaxation pathway, directly related to the ion concentration and separation distance.[17] At high Tb^{3+} concentrations, the excited electrons can cross-relax from the $^5D_3$ to the $^5D_4$ level.[17] The process is associated with the energy transfer from the Tb^{3+} $^5D_3$ excited state to the ground state ion:[17,18]

$$Tb^{3+}\ (^5D_3) + Tb^{3+}\ (^7F_4) \rightarrow Tb^{3+}\ (^5D_4) + Tb^{3+}\ (^7F_0)$$

As discussed earlier, for lower Tb^{3+} concentrations, the cross-relaxation probability is decreased, which promotes the $^5D_3 \rightarrow {^7F_J}$ transitions.[17] The effect is more pronounced at low concentrations and is typically noted in cathodoluminescence spectra but not found in the case of photoluminescence.[17,19] The blue emission has been correlated with Tb^{4+} ions, substituting the Zr^{4+} ion positions. Upon the electron (e^-) impact from the beam during cathodoluminescence measurements, Tb^{4+} ions can transiently be excited to the $(Tb^{3+})^*$ state which results in the formation of emissive centers[19]:

$$Tb^{4+} + e^- \rightarrow (Tb^{3+})^* \rightarrow Tb^{4+} + h\nu_{luminescence}\ (^5D_3, {^5D_4} \rightarrow {^7F_J})$$

In the CL spectra collected for $ZrO_2{:}Tm^{3+}$ (Fig. S4 (c))., significant narrowing and relative increase of the $^1D_2 - {^3F_4}$ transition intensity is observed for the samples annealed at 1200 °C for one and three hours. In addition, weak $^1D_2 \rightarrow {^3H_6}$ and $^1G_4 \rightarrow {^3F_4}$ transitions are observed after treatment at 1200 °C for 1 h. The latter transition is not discernible after the treatment for 3 h. Finally, the CL spectra collected for triply-doped microstructures ($ZrO_2{:}Eu^{3+}$, Tb^{3+}, Tm^{3+}) are discussed (Fig. S4 (d)). A notable difference concerns the suppression of the characteristic $^5D_0 \rightarrow {^7F_J}$ (J=1,2,3,4) transitions of Eu^{3+} for both samples annealed at 1200 °C for one and three hours. Similarly, the Tb^{3+} $^5D_4 \rightarrow {^7F_5}$ signal intensity is significantly decreased. The Tm^{3+} $^1D_2 \rightarrow {^3F_4}$ transition, and Tb^{3+} $^5D_3 \rightarrow {^7F_6}$ and $^5D_3 \rightarrow {^7F_4}$ transitions can be distinguished. Similar trends can be observed for $ZrO_2{:}Eu^{3+}$, Tb^{3+}, and Tm^{3+} (8 mol % of lanthanides in total) synthesized using a sol-gel method and a polymeric matrix by Lovisa *et al.*[20] The region from 4.1 eV to ≈ 2.4 eV (or ≈ 300 – 517 nm) appears to contain a buried broadband feature, which may be the sum of different contributions. As the intensity of $Eu^{3+}{^5D_0} \rightarrow {^7F_J}$ (J=1,2,3) and Tb^{3+} $^5D_4 \rightarrow {^7F_5}$ transitions are affected, we postulate that the observations may be correlated to the reduction of Eu^{3+} to Eu^{2+}, the oxidation of Tb^{3+} to Tb^{4+}. The reduction of Eu^{3+} and oxidation of Tb^{3+} has previously been observed under similar conditions in, e.g., $SrAl_2Si_2O_8$[21] or may be associated with impurities.[22] As presented, the thermal treatment opens the opportunities for further tuning the optical properties of the triply-doped microstructures ($ZrO_2{:}Eu^{3+}$, Tb^{3+}, Tm^{3+}), which may allow the realization of complex emissions in colors other than primary or white, presented for structures annealed at 600 °C for 1 h. From a different perspective, the use of lower temperatures is attractive due to the energy use concerns and permits for the integration across different manufacturing processes.

References

1 T. Laetsch and R. Downs, in *Abstracts from the 19th General Meeting of the International Mineralogical Association*, Kobe, Japan, 2006.

2 Y. S. Vidya, K. S. Anantharaju, H. Nagabhushana, S. C. Sharma, H. P. Nagaswarupa, S. C. Prashantha, C. Shivakumara and Danithkumar, *Spectrochim. Acta - Part A Mol. Biomol. Spectrosc.*, 2015, **135**, 241–251.

3 L. X. Lovisa, J. Andrés, L. Gracia, M. S. Li, C. A. Paskocimas, M. R. D. Bomio, V. D. Araujo, E. Longo and F. V. Motta, *J. Alloys Compd.*, 2017, **695**, 3094–3103.

4 A. L. Patterson, *Phys. Rev.*, 1939, **56**, 978–982.

5 K. He, N. Chen, C. Wang, L. Wei and J. Chen, *Cryst. Res. Technol.*, 2018, **53**, 1700157.

6 G. Pezzotti and A. A. Porporati, *J. Biomed. Opt.*, 2004, **9**, 372.

7 X. Zhao and D. Vanderbilt, *Phys. Rev. B*, 2002, **65**, 075105.

8 A. P. Naumenko, N. I. Berezovska, M. M. Biliy and O. V Shevchenko, *Phys. Chem. Solid State*, 2008, **9**, 121–125.

9 J. Winczewski, M. Herrera, C. Cabriel, I. Izeddin, S. Gabel, B. Merle, A. Susarrey Arce and H. Gardeniers, *Adv. Opt. Mater.*, 2022, **10**, 2102758.

10 C. Tiseanu, B. Cojocaru, V. I. Parvulescu, M. Sanchez-Dominguez, P. A. Primus and M. Boutonnet, *Phys. Chem. Chem. Phys.*, 2012, **14**, 12970–12981.

11 C. Tiseanu, V. I. Parvulescu, B. Cojocaru, K. Pemartin, M. Sanchez-Dominguez and M. Boutonnet, *J. Phys. Chem. C*, 2012, **116**, 16776–16783.

12 B. Li, D. Yu and S. Zhang, *Phys. Rev. B - Condens. Matter Mater. Phys.*, 1999, **59**, 1645–1648.

13 J. Zhou, R. Lei, H. Wang, Y. Hua, D. Li, Q. Yang, D. Deng and S. Xu, *Nanophotonics*, 2019, **8**, 2347–2358.

14 A. N. Bugrov, R. Y. Smyslov, A. Y. Zavialova, G. P. Kopitsa, T. V. Khamova, D. A. Kirilenko, I. E. Kolesnikov, D. V. Pankin, V. A. Baigildin and C. Licitra, *Crystals*, 2020, **10**, 1–19.

15 A. Jones, in *Practical Raman Spectroscopy - An Introduction*, ed. P. Vandenabeele, John Wiley & Sons, Ltd, Chichester, UK, 1st edn., 2013, pp. 1–38.

16 A. J. Silversmith, D. M. Boye, K. S. Brewer, C. E. Gillespie, Y. Lu and D. L. Campbell, *J.*

Lumin., 2006, **121**, 14–20.

17 H. A. A. Seed Ahmed, O. M. Ntwaeaborwa, M. A. Gusowski, J. R. Botha and R. E. Kroon, *Phys. B Condens. Matter*, 2012, **407**, 1653–1655.

18 Y. Wang, Z. Zhu, S. Ta, Z. Cheng, P. Zhang, N. Zeng, B. A. Goodman, S. Xu and W. Deng, *Crystals*, , DOI:10.3390/cryst12081081.

19 M. Zawadzki, D. Hreniak, J. Wrzyszcz, W. Miśta, H. Grabowska, O. . Malta and W. Stręk, *Chem. Phys.*, 2003, **291**, 275–285.

20 L. X. Lovisa, V. D. Araújo, R. L. Tranquilin, E. Longo, M. S. Li, C. A. Paskocimas, M. R. D. Bomio and F. V. Motta, *J. Alloys Compd.*, 2016, **674**, 245–251.

21 Y. Pan, W. Wang, Y. Zhu, H. Xu, L. Zhou, X. Liu and L. Li, *J. Alloys Compd.*, 2018, **769**, 932–939.

22 S. Liu, G. Zhao, W. Ruan, Z. Yao, T. Xie, J. Jin, H. Ying, J. Wang and G. Han, *J. Am. Ceram. Soc.*, 2008, **91**, 2740–2742.
